# A Treatise on Venereal Diseases

**Published:** 1853-12

**Authors:** 


We have received the work which is the subject of the ensuing notice. Not having time for its perusal, we defer the task of its review to a Boston cerrespondent, who has kindly furnished us the following critique:

S. B. H.

*A Treatise on Venereal Diseases.* By John Hunter, F. R. S. With copious additions by Dr. Philip Ricord, Surgeon of the Hospital du Midi, Paris, etc. Edited, with notes, by Freeman J. Bumstead, M. D., Physician to the North-Western Dispensary, New York. Philadelphia: Blanchard & Lea. 1853. 1 vol. 8vo.

Within the last year or two the profession has been greatly benefited by additions to its syphilographic literature. In Europe the advance of this portion of our science has been rapid, and few of our profession come home from their continental tour unaffected by what they have seen and heard in this department, especially if they have had the good fortune to be listeners to Ricord's eloquent lessons in the courtyard of the Hospital du Midi. The increase of prostitution and syphilis in this country, coincident with its rapid march in civilization, brings with it the growing necessity for a more accurate knowledge of the means for eradicating its fatal and disastrous effects; and it must be acknowledged that in this respect we are far behind our brethren of *outre mer.* The writings of Maissonneuse and Montanier, of Puche, Cullerier and Vidal (de Cassis) in France; of Acton, Meric and Egan, in Great Britain; and the translations of Ricord's letters in our own country, all of whom have issued their works within three years, show the interest already existing, and the hiatus that was to be filled in this part of surgical literature. For a clear and concise review of the present state of what, since the syphilization excitement, we must now call *legitimate* syphilis, can anything be more promising than the united names of Ricord and Hunter?

We hold that Dr. Bumstead has done great service in translating this work, which is a new edition, first from the cabinet of the Rue de Toumon, one that has never before been presented to the American reader, and a better translation of an author, than whom, in his vivid and fiery style, flashing and rapid as the wheels of his "*coupe*," in which the "notes" were actually written as Ricord drove his daily round of visits, none could be more difficult to render in sober English, we think could hardly be offered for criticism. This volume, with its clear type and complete index, constitutes a manual of easy reference and of rich contents. The venereal disease in every radiation of its intricate divergencies from its primary source, is thoroughly and carefully considered, not only by Hunter and his annotator, Babbington, but by Ricord himself. The American editor is not merely a translator of these additions to the original text of Hunter, but has himself brought out all that can be found in other modern authors, especially those whose names we have above mentioned; these he has analyzed and compared with his own authors, reconciled their differences or exposed their inconsistencies, and quoted their formula and prescriptions.

The book as a whole is the most complete treatise on the subject as it acactually stands, now to be found. Ricord's notes constitute one-third of the whole work, and the additions of the editor in this country are not inconsiderable. Himself recently a student at the Hospital des Veneriens, he has gone to his work with all the zeal that the teachings of such a master must inspire. The work should be read by all students as well as practitioners, and to it in all confidence may they turn sure to find the counsel that they desire.

For sale by DERBY, ORTON & MULLIGAN.

